# Adolescence is an opportunity for farm injury prevention: A call for better age-based data disaggregation

**DOI:** 10.3389/fpubh.2022.1036657

**Published:** 2022-10-20

**Authors:** Amy E. Peden, Tich Phuoc Tran, Dennis Alonzo, Catherine Hawke, Richard C. Franklin

**Affiliations:** ^1^School of Population Health, UNSW Sydney, Kensington, NSW, Australia; ^2^College of Public Health, Medical and Veterinary Sciences, James Cook University, Townsville, QLD, Australia; ^3^School of Science, University of New South Wales, Canberra, ACT, Australia; ^4^School of Education, UNSW Sydney, Kensington, NSW, Australia; ^5^School of Rural Health, University of Sydney, Orange, NSW, Australia

**Keywords:** injury, agriculture, adolescence, occupation, recreation, drowning, quad bike, transport

## Introduction

Injury is a leading cause of mortality and injury-related morbidity, which can have lifelong impacts on physical and mental health, as well as on an individual's and family's economic livelihood (1).

Transport and unintentional injuries are the leading cause of death for adolescents 10–24 years of age, with more lives lost than communicable or non-communicable diseases, nutritional or maternal health causes or self-harm (2). Predominantly, in the injury prevention arena, there is a tendency to focus on young (especially under 5 years) children and therefore, despite the persistently high injury burden among adolescents, there has been limited research on, and evaluation of, the prevention of injury-related harms among adolescents (3).

## Farm injury and its prevention

For adolescents, the farm environment poses a unique risk of injury as it is often a home, a workplace and a place for recreation with adolescents moving between these activities regularly (4). Farms are environments full of hazards such as noise (5), electricity (6), vehicles (7) including off-road motorcycles (8), quad-bikes (9) and utilities, agricultural machinery such as tractors and augers (10), as well as animals (11), plants and the broader environment. Additionally, living and working on farms exposes people to greater risk of injury from large farm animals including horses, as well as drowning risk due to unfenced natural water bodies (12).

In Australia, agriculture as an industry averages 82 non-intentional farm injury deaths per year between 2003 and 2006 (13). In New South Wales, Australia's most populous state, there was an average annual rate of 17.3 work-related deaths on farms per 100,000 people working in agriculture between 2001 and 2015 (14). With respect to hospitalized farm injury in Australia, between 2010–11 and 2014–15, there were a total of 21,999 farm injury related hospitalizations, with greater burden among males and in inner and outer regional areas (12).

Internationally, agriculture is one of the most dangerous industries in which to work and this is likely to increase with changes in climate, markets, transport systems and cost, staffing issues and farming practices (15). Evidence out of the United States of America indicates a child dies in an agriculture-related incident every 3 days, with transportation, machinery and contact with animals the leading mechanisms of injury (16). Agriculture has a wide range of hazards such as heavy machinery, chemicals and animals. In non-western countries, varying risks are present such as the risk of injury to toes and figures from hand tools for preadolescent agricultural workers in rural India (17).

**Figure 1:**
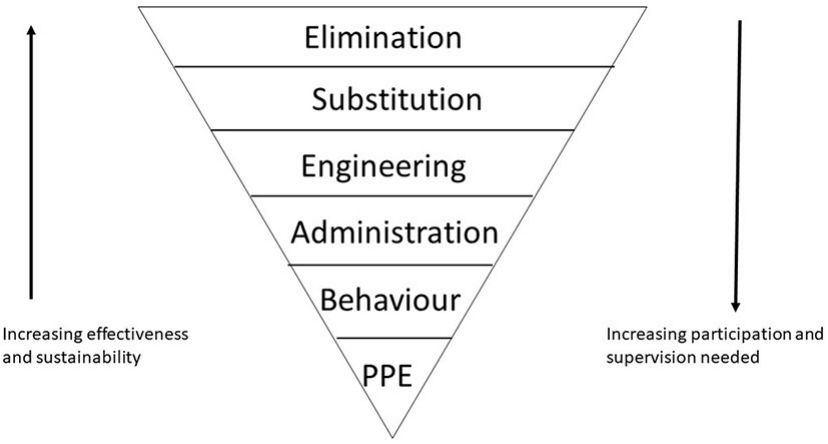
The hierarchy of control [modified from Franklin and Scarr (19)].

Work in and around the farm often consists of tasks which over the course of a day can be extremely varied, each with their own risks. To address this in Australia the hierarchy of control, a framework of measures ranked on level of health and safety protection and reliability of control measures, is now embedded in legislation and provides a framework on which to address safety (18). A version of the hierarchy of control, as modified from Franklin and Scarr (19) can be found in Figure 1.

Farm injury risk can differ by age, with children and adolescents on farms having been identified as being at increased risk of injury (4). This injury risk arises as young people are often exposed to risks and hazards not normally present in a home environment and may have on-farm responsibilities which can lead to injury (4). Additionally, in Australia, a third of all child farm-related fatalities were among farm visitors (20). Such risks persist and, as such, fatal farm incidents among children <15 years in Australia have remained largely unchanged between 2001 and 2019, indicating a lack of progress on preventable deaths of children on farms (20).

## Lack of age-based data disaggregation for adolescent farm injury in Australia

While Australia is fortunate to have regular and reliable data capture and collection of both fatal and non-fatal injury, including on farms, farm injury data on both deaths and hospitalizations are currently presented across two broad age bands: 0–14 years and 15+ years. Adolescence, which is defined as 10–24 years to reflect adolescent growth and popular understandings of this phase of life (21), therefore spans both age groups, making it difficult to derive age-specific injury risks for this cohort. This leads to the need for data disaggregation which involves separating collected information into smaller segments to discover useful trends and patterns.

So why is age disaggregated data on farm injury-related mortality and morbidity necessary? The development of effective injury prevention interventions must be evidence-informed. This includes an understanding of how injury risk differs for different age groups. Leading injury control approaches, such as the Haddon's Matrix and the Public Health approach (22) include the identification of risk factors (such as age) to inform injury prevention efforts.

Generally speaking, injury does not occur among, or affect, different population subgroups in the same manner. In particular, injury risk for adolescents on farms is likely to differ significantly from young children and is likely to differ again from adults. Within reported farm injury-related fatalities for the 0–14 years age band in Australia, we see a concentration of drowning fatalities among children 0–4 years in farm dams, tanks and water troughs (20) the causal factors for which—low swimming ability, lack of adult supervision and barriers to water (23)—are unlikely to be of relevance to adolescents. Childhood farm injury mechanisms also include trail bikes, horse-related injuries and burns (9, 24, 25). By contrast, injuries related to the operation of farm machinery more often affect adult and older adult operators of such machinery who are injured while working (12, 14). However, quadbike injury risk is one that may span age—with adolescents likely to feature among quad bike injury statistics as both operator and passenger, and injury risk persisting into adulthood (14).

If undertaken in a developmentally appropriate way (26), the adolescent years represent an important opportunity to provide farm safety education with the goal of intervening to change behavior (27). This may be *via* the school system, as well as presenting an opportunity to provide education to those who may not yet have been exposure to farm injury prevention information, either in their younger years or *via* their parents (28). With respect to farm injury prevention, adolescence is an optimal time to continue the safety conversation and can reinforce lessons from earlier years, which may then be applied as adolescents transition into the workforce (29).

To ensure the safety of adolescents on farms there is a need to ensure that the messages they receive are relevant, come from their peers and people they respect (including parents and people in positions of authority), are linked to the activities they are doing on farms, and are evidenced based (30). Safety information must also be followed up where possible with training (both informal and formal) and further activities with reinforce good safety practices, including legislation (30). Coaching is one approach that has been proposed to help with both improving safety practices, but also business practices more widely (31). Linking this to educational opportunities and a greater understanding of adolescent human factors would then help to nudge the culture toward being safer while also being productive (32, 33). School curriculum is one of the best avenues for providing training to adolescents (34). Developing and implementing interventions linked to curriculum standards have been proven to enhance their positive attitudes and result in higher intent to change their risky behavior (35). Universal health education on farming injury risk, rather than focusing simply on farming communities, is also warranted given a third of all child farm-related fatalities were among farm visitors, not farm residents (20).

Prior work to establish a prevention strategy for children in Australia has found that establishing a strong evidence base led to a tightly focused strategy (36). To inform the development of farm injury preventive interventions specific to adolescents, we call for improved data disaggregation to derive risk factors and evaluate injury prevention interventions specific to farm dwelling and working adolescents. Better data disaggregation on farm injury risk for adolescents will result in better understanding of the issue, including how risk varies by age, and therefore improved prevention interventions. A reversal in the neglect shown for adolescent injury prevention (2), including those injuries which occur on farms and in regional areas (37), would yield significant benefits, including the triple dividend (38) of reduced injury risk during adolescents, for adults working on farms and into the next generation of children living, working and recreating on farms.

Although there may be challenges around disaggregation of data in countries like Australia due to small sample sizes, this data could be made available upon request to those with approval to handle such sensitive data for the purposes of injury prevention. Another potential solution would be the reporting of disaggregated age group data for farm injuries over a greater period of time—i.e., 10 years or more to allow for more meaningful disaggregation while addressing sample size concerns. Similarly, a global repository of adolescent farm injury data would allow for secondary analysis of de-identified data and consistent reporting of age groups which varies widely for the adolescent age group (12, 13, 16).

In short, we firmly believe that sufficient disaggregation of age-based data enables more effective interventions and supports policies and strategies to address challenges in adolescent farm injury.

## Author contributions

AP and RF conceptualized the study. AP wrote the first draft with assistance from RF. TT, DA, and CH critically revised the draft. All authors agree to be accountable for the content of the work. All authors contributed to the article and approved the submitted version.

## Funding

This work was funded by the National Farm Safety Education Fund: Improving Farm Safety Practices (Grant ID: 4-GASG0RA). AP was funded by a National Health and Medical Research Council (NHMRC) Emerging Leadership Fellowship (Grant ID: APP2009306).

## Conflict of interest

The authors declare that the research was conducted in the absence of any commercial or financial relationships that could be construed as a potential conflict of interest.

## Publisher's note

All claims expressed in this article are solely those of the authors and do not necessarily represent those of their affiliated organizations, or those of the publisher, the editors and the reviewers. Any product that may be evaluated in this article, or claim that may be made by its manufacturer, is not guaranteed or endorsed by the publisher.
